# Exploring the rising death rates among older US citizens with heart failure and sepsis: Need for health-care policy reform

**DOI:** 10.1016/j.ijcrp.2025.200428

**Published:** 2025-05-11

**Authors:** Abdul Ahad, Eeshal Fatima, Adeena Jamil, Obaid Ur Rehman, Irfan Ullah, Raheel Ahmed, Bernardo Cortese, Michael E. Hall, Mamas A. Mamas

**Affiliations:** a Kabir Medical College, Gandhara University, Peshawar, Pakistan; b Emergency Department, Northwest General Hospital and Research Centre, Peshawar, Pakistan; c Department of Medicine, Services Institute of Medical Sciences, Lahore, Pakistan; d Department of Medicine, Dow International Medical College, Dow University of Health Sciences, Karachi, Pakistan; e Department of Internal Medicine, Khyber Teaching Hospital, Peshawar, Pakistan; f National Heart and Lung Institute, Imperial College London, UK; g University Hospitals Harrington Heart and Vascular Institute, Case Western Reserve University, Cleveland, OH, USA; h DCB Academy, Milano, Italy; i Fondazione Ricerca e Innovazione Cardiovascolare, Milano, Italy; j Department of Medicine, University of Mississippi Medical Center, Jackson, MS, USA; k Keele Cardiovascular Research Group, Keele University, Stoke on Trent, UK; l National Institute of Health and Care Research (NIHR), Birmingham Biomedical Research Centre, UK

**Keywords:** Heart failure, Sepsis, CDC WONDER, United States

## Abstract

**Background:**

Heart failure (HF) patients, often multimorbid and immunocompromised, are particularly vulnerable to sepsis, which increases their risk for mortality. It is therefore crucial to understand the mortality patterns in older adults (≥65) with HF and sepsis for improving care strategies in the United States.

**Methods:**

A cross-sectional evaluation of death certificates from the Centers for Disease Control and Prevention's Wide-Ranging Online Data for Epidemiologic Research (CDC WONDER) was conducted to examine HF and sepsis mortality trends. For demographic and geographic subgroups, age-adjusted mortality rates (AAMR) per 100,000 older individuals, alongside annual percent change (APC) in AAMR with 95 % confidence intervals (CI), were derived.

**Results:**

Throughout 1999 to 2019, 250,115 deaths associated with HF and sepsis were recorded among older adults, the majority being reported in medical facilities. The overall AAMR escalated from 28.8 in 1999 to 33.7 in 2019. AAMRs were more pronounced in men (33.9) than in women (25.5). Non-Hispanic (NH) Black/African American individuals exhibited the highest AAMRs (42.4). At the same time, NH Asian/Pacific Islanders reported the lowest (18.9). AAMRs were higher in non-metropolitan areas (31.4) and the Southern region (31.1). State-level disparities revealed the highest AAMRs in Mississippi (45.4) and the lowest in Arizona (12.1).

**Conclusion:**

Mortality from HF and sepsis has increased over the last 20 years, with NH Black/African individuals demonstrating the greatest increases. Future studies should investigate the mechanisms underlying this increase, particularly focusing on the factors that drive disparities, to develop targeted interventions for vulnerable groups.

## Highlights

• Coexisting heart failure (HF) and sepsis in older adults leads to higher mortality.
• The rising fatality rate since 2012 is worrisome across the 20 years of the study.
• Older men and non-Hispanic Black populations reported the highest fatality rates.
• Geographically, the South and rural localities experienced the highest death rates.
• Understanding of mortality trends will help deliver focused healthcare to at-risk communities.

## Graphical abstract

**Figure:**
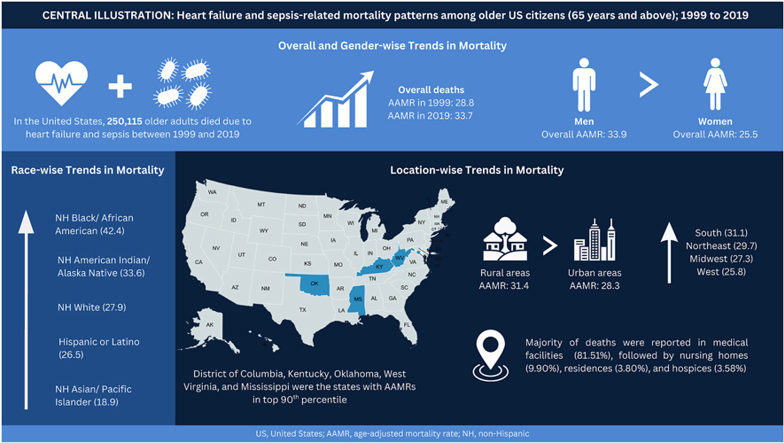

## 1 Introduction

Approximately 6.7 million individuals in the United States (US) suffer from heart failure (HF), a chronic, progressive condition [1]. With a five-year mortality rate of around 50 %, it is one of the most frequent reasons for hospitalization, especially in elderly patients [2]. In addition to its impact on the cardiovascular system, HF is associated with substantial non-cardiovascular mortality [3]. Given the comorbid conditions endured by patients with HF, they are at increased risk for non-cardiovascular complications [4,5]. Influences of immune dysfunction and more time spent in the healthcare environment contribute even more to heightened mortality risks from infections, especially sepsis [6,7].

Infections are commonly observed as a cause of acute decompensation in patients with chronic HF, with sepsis and septic shock being responsible for approximately 25 % of HF-related deaths [6]. The pathophysiology of HF complicated by sepsis is a complex process primarily due to the release of different cytokines, mitochondrial dysfunction, and tissue hypoxia, which ultimately leads to injury and death of the cardiac myocytes [8]. It is difficult to treat because of the conflicting therapeutic strategies in HF and sepsis. Although sepsis management includes aggressive crystalloid fluid resuscitation and using vasopressors to provide hemodynamic support, conventional HF strategies focus on decreasing pre- and afterload [9]. Consequently, patients with HF and sepsis have high mortality rates as well as a very high probability of being admitted to the intensive care unit (ICU).

To gain a deeper understanding of mortality trends in these patients, we analyzed death certificate data of older US citizens, focusing on demographic and regional mortality patterns from 1999 to 2019 to identify vulnerable groups.

## 2 Material and methods

### 2.1 Research setting and target population

We utilized the National Center for Health Statistics (NCHS) death certificate data provided via the Centers for Disease Control and Prevention's Wide-Ranging Online Data for Epidemiologic Research (CDC-WONDER) Database [10] to assess the HF and Sepsis-associated fatality within the patient population of 65 years and above throughout 1999 to 2019. The Multiple Cause of Death Public Use Record and the International Statistical Classification of Diseases and Related Health Problems-10th Revision (ICD-10) codes A40 and A41 for sepsis and I11.0, I13.0, I13.2, and I50 for HF were used to extract data [11,12]. Moreover, as a result of a substantial rise in deaths due to COVID-19 between 2019 and 2020, we restricted our analysis to 2019 and reported the overall mortality separately for patients with co-existing HF and sepsis from 2020 to 2022, to account for COVID-19 as a potential confounder.

### 2.2 Study design

We extracted data for a range of demographic traits that comprised gender, race and ethnicity, geographical distribution including census region and states, and urban-rural classification. The individuals were categorized into the following racial and ethnic groups: Non-Hispanic (NH) American Indian or Alaska Native, NH Asian or Pacific Islander, NH Black or African American, NH White, and Hispanic or Latino. The regions were subdivided, under U.S. Census Bureau definitions, into Northeast, Midwest, West, and South. The Urban-Rural Classification Scheme from the NCHS was employed to outline urban (large metropolitan localities having a population of ≥1 million, and medium/small metropolitan localities comprising a population of 50,000–999,999) and rural (comprising a population of less than 50,000) areas based on the 2013 U S. census classification [13].

### 2.3 Statistical evaluation

To inspect HF and sepsis-associated death patterns in the US, we computed crude and age-adjusted mortality rates (AAMRs) per 100,000 population along with associated 95 % confidence intervals (CIs). AAMR accounts for differences in the age distribution of the population, enabling data comparison. We calculated AAMRs by adjusting the number of deaths to the US population data from the year 2000, and crude mortality rates (CMRs) were established by obtaining the number of deaths and then dividing them by the corresponding US population of that year [14]. To analyze age-adjusted mortality trends, the Joinpoint Regression Program (Joinpoint version 5.0.2, National Cancer Institute) was implemented [15]. The program employs serial permutation tests to assess repeated time trends and identifies the inflection point where the mortality rate change is statistically significantly different. It then calculates the average annual percent change (AAPC) and annual percent change (APC) for each period in the AAMR, along with associated 95 % CIs. The confidence interval for APC/AAPC/Tau was obtained through the empirical quantile method. An APC estimate was noted as indicating an increase or decrease if the slope of the trend significantly diverged from zero; otherwise, the trend was considered stable. A statistically significant trend change was defined by a P value of less than 0.05.

### 2.4 Approval of standard protocol, patient registrations, and consents

Since the analysis was based upon the utilization of de-identified data, provided for public use by the government, the research was therefore deemed exempt from acquiring approval by an institutional review board.

## 3 Results

Over a period spanning 1999 to 2019, 250,115 older adults received a diagnosis of HF and sepsis on their death certificate **(**Supplemental Table A1**)**. Regarding the locations where the deaths were recorded, data were accessible for only 249,484 fatalities, with the majority of them being reported in medical facilities (81.51 %), followed by nursing homes (9.90 %), residences (3.80 %), and hospices (3.58 %) **(**Supplemental Table A2**)**.

### 3.1 Overall mortality pattern for HF and sepsis combined, HF alone, and sepsis alone

**Fig. 1:**
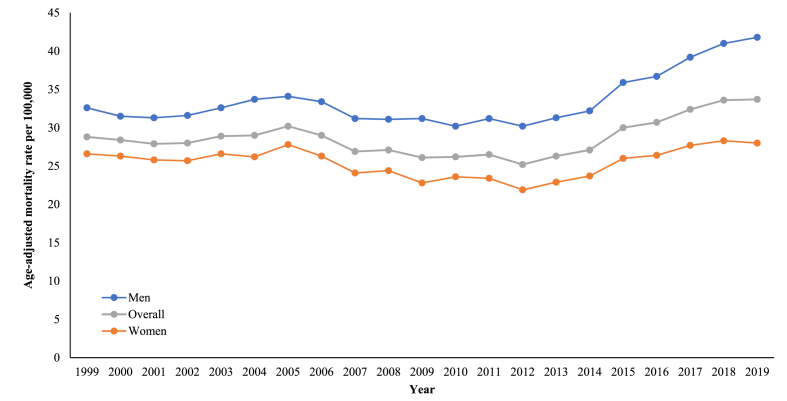
Patterns related to heart failure and sepsis-associated age-adjusted death rates per 100,000 population, categorized by overall and sex, amongst older US citizens, across the years 1999–2019.

The overall AAMRs for older adults with HF and sepsis increased from 28.8 in 1999 to 33.7 in 2019 (AAPC with 95 % CI value: 1.06; 0.72 to 1.57) **(**Supplemental Table A3**)**. After an initial period of stability, AAMRs exhibited a steady downward trend between 2005 and 2012 (APC with 95 % CI value: −2.06; −6.85 to 2.91). A reversal in trend was noted with AAMRs substantially rising for the remainder of the study duration (APC with 95 % CI value: 4.72; 3.20 to 7.37) (Fig. 1, Supplemental Table A9).

The overall mortality for HF alone decreased from 793.1 in 1999 to 680.6 in 2019 (AAPC with 95 % CI value: −0.80; −0.91 to −0.68) **(**Supplemental Table A3**)**. The AAMRs dropped from 1999 to 2012 (APC with 95 % CI value: −2.27; −2.47 to −2.08); however, the trend changed with the death rates significantly rising until 2019 (APC with 95 % CI value: 2.00; 1.49 to 2.61) **(**Supplemental Table A9**)**.

Similarly, in the case of sepsis alone, the overall death rate decreased from 306.0 in 1999 to 288.5 in 2019 (AAPC with 95 % CI value: −0.28; −0.48 to −0.10) **(**Supplemental Table A3**)**. The mortality trend declined initially between 1999 and 2012 (APC with 95 % CI value: −0.48; −0.85 to −0.25), trailed by a substantial rise until 2017 (APC with 95 % CI value: 1.66; 0.90 to 3.36) and then an eventual decline for the remaining years (APC with 95 % CI value: −3.67; −5.79 to −1.23) **(**Supplemental Table A9**)**.

### 3.2 Mortality patterns related to concurrent HF and sepsis concerning demographic subgroups

#### 3.2.1 Sex

Men had consistently higher overall death rates (33.9) throughout the study duration in comparison to women (25.5) **(**Supplemental Table A4**)**. Mortality remained relatively stable for both sexes until 2005, then a notable decline in death rates in women was detected (APC with 95 % CI value: −2.50; −6.24 to −1.33), while a steady decline in the case of men was identified (APC with 95 % CI value: −1.46; −5.93 to 6.57), until 2012. Both sexes then experienced a significant upsurge in death rates, although more pronounced in the case of men, up until 2019 (APC for men with 95 % CI value: 5.17; 3.08 to 7.97; APC for women with 95 % CI value: 3.97; 2.68 to 5.91) (Fig. 1, Supplemental Table A9).

#### 3.2.2 Race and ethnicity

**Fig. 2:**
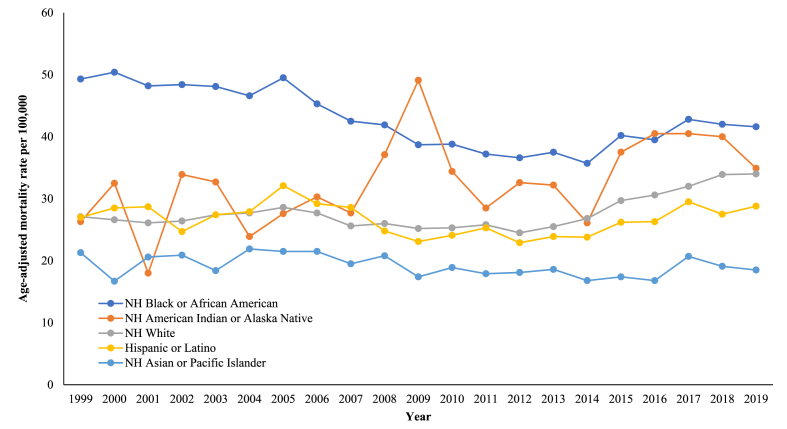
Patterns related to heart failure and sepsis-associated age-adjusted death rates per 100,000 population, categorized by race and ethnicity, amongst older US citizens, across the years 1999–2019.

Disparities within this demographic subgroup revealed the most elevated overall death rates in the case of NH Black or African American populations (42.4) and then NH American Indian or Alaska Native populations (33.6), which were closely followed by NH White (27.9) and Hispanic or Latino (26.5) populations. In contrast, NH Asian or Pacific Islander populations displayed the least overall fatality (18.9) **(**Supplemental Table A5**)**. Death rates remained relatively stable for NH Black and African American and NH White populations until 2005, while steadily increased for Hispanic or Latino populations until 2006 (APC with 95 % CI value: 1.80; −0.17 to 8.48). This was followed by a significant decrease for NH Black and African populations until 2011 (APC with 95 % CI value: −4.50; −8.38 to −3.02) and Hispanic or Latino populations until 2009 (APC with 95 % CI value: −8.92; −12.10 to −1.96). NH White populations, on the other hand, underwent a steady decline between 2005 and 2012 (APC with 95 % CI value: −1.64; −5.84 to 3.81). The death rates then significantly increased for the three racial and ethnic groups throughout the remaining of the research duration (APC with 95 % CI value for NH Black or African American: 2.09; 1.10 to 3.52; APC with 95 % CI value for NH White: 5.19; 3.66 to 7.57; APC with 95 % CI value for Hispanic or Latino: 2.34; 1.23 to 5.04). Throughout the study time, NH American Indian or Alaska Native populations experienced a rise in fatality (APC with 95 % CI value: 1.63; 0.23 to 3.49), while on the flip side, NH Asian or Pacific Islander populations showed a relatively stable pattern. (Fig. 2, Supplemental Table A9).

#### 3.2.3 Geographical variations

**Fig. 3:**
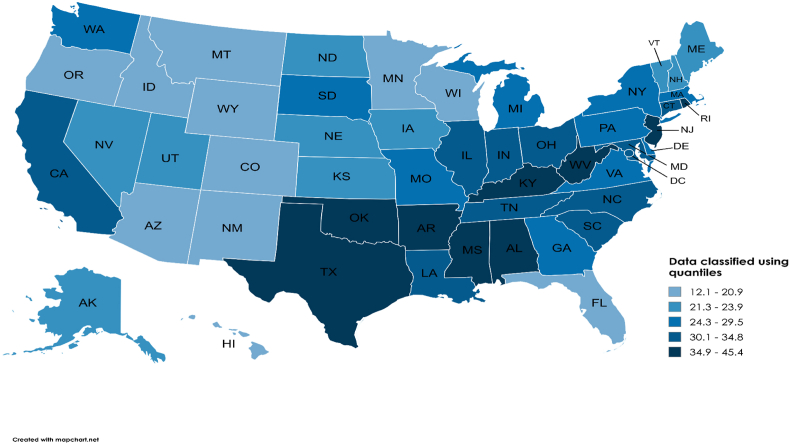
Variations in heart failure and sepsis-associated age-adjusted death rates per 100,000 population, categorized by states, amongst older US citizens, across the years 1999–2019. ∗Map lines delineate study areas and do not necessarily depict accepted national boundaries.

Variations in overall mortality among the states were obvious, with the highest death rates reported in Mississippi (45.4) and the lowest in Arizona (12.1). District of Columbia, Kentucky, Oklahoma, West Virginia, and Mississippi were the states comprising AAMRs that fell in the top 90th percentile range, with mortality rates approximately three folds higher than the states included in the bottom 10th percentile namely Wyoming, Montana, Hawaii, Florida, and Arizona (Fig. 3, Supplemental Table A6).

Regionally, fatality rates peaked in the Southern region (31.1), followed closely by the Northeastern (29.7), the Midwestern (27.3), and the Western (25.8) regions **(**Supplemental Table A7**)**.

**Fig. 4:**
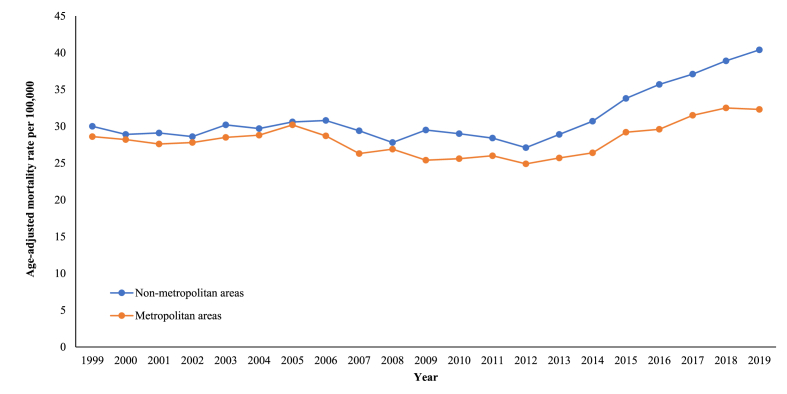
Patterns related to heart failure and sepsis-associated age-adjusted death rates per 100,000 population, categorized by urban-rural classification, among older US citizens, across the years 1999–2019.

Non-metropolitan areas had higher overall fatality rates (31.4) when compared to metropolitan areas (28.3) **(**Supplemental Table A8**)**. After a period of stability, the AAMRs in metropolitan areas steadily dropped between the period 2005 and 2012 (APC with 95 % CI value: −2.16; −6.65 to 1.81). On the flip side, non-metropolitan areas experienced a period of relative stability in mortality from 1999 to 2012. Thereafter, both localities exhibited a noteworthy rise in death rates for the remainder of the period (APC with 95 % CI value for metropolitan areas: 4.40; 2.89 to 7.02; APC with 95 % CI value for non-metropolitan areas: 5.41; 4.09 to 7.38) (Fig. 4, Supplemental Table A9).

### 3.3 Overall mortality patterns concerning coexisting HF and sepsis for the year intervals 2020 to 2022

An overall AAMR of 38.8 was noted for individuals diagnosed with coexisting HF and sepsis, with a total death count of 61,472 during this period. The AAMRs rose from 36.5 in 2020 to 40.1 in 2021 and were finally observed to be at 39.9 in 2022.

## 4 Discussion

The findings reported in our study provide important insights regarding mortality patterns related to HF and sepsis in older US citizens during a 20-year timeframe. We observed a relatively stable mortality rate from 1999 to 2005, which was then followed by a subsequent decrease until 2012. This was followed by a period of upward trend until 2019. The rising trend of mortality continued in the separate evaluation of the 2020 to 2022 period. Men, throughout the research timeframe, experienced higher fatality rates than women. With regards to death rates within the racial groups, NH Black exhibited the highest mortality, in contrast to the lowest being reported for NH Asian or Pacific Islanders. We also studied the geographic and regional variations in the trends, with the highest and lowest fatality rates observed in the Southern and Western regions, respectively, with an almost 3-fold difference in AAMR amongst different states.

Both sepsis and HF remain predominant causes of mortality among critically ill patients, with a notably higher incidence observed in the elderly population [16,17]. Recent definitions of sepsis acknowledge the significance of myocardial depression by incorporating a low cardiac index or echocardiographic evidence of cardiac dysfunction as key diagnostic criteria for severe sepsis [16]. Studies indicate that infections account for more than 25 % of acute HF cases [18]. Additionally, in patients with severe sepsis and septic shock, chronic HF has been recognized as an independent risk factor for increased fatality [19].

The findings of our study identified a consistently slowly rising trend of mortality due to HF and sepsis ever since 2012, on a background of relative stability before that. This can be attributed to the fact that both sepsis and HF have significant morbidity and mortality [16,17], which becomes more pronounced with advancing age. For example, a previous study reported that the mortality rate in patients with cardiac insufficiency and sepsis can be extremely high, potentially reaching 90 % [20]. An intensive-care over nations (ICON) audit reported that the mortality rate of sepsis can be as high as 25.8 % and 35.3 % in the ICU and hospital, respectively [21]. Another study documented an increase in the annual incidence of severe sepsis from 1993 to 2003 in the US, which has recently reached 132 cases per 100,000 population, with a mortality rate approaching 50 % [22]. According to a study, infections in the elderly are not only more frequent but also more severe, often posing life-threatening risks. The heightened susceptibility to infections in this population can be attributed to a combination of epidemiological factors, immunosenescence, malnutrition, and numerous age-related physiological and anatomical changes [23]. The incidence of HF progressively increases with age, reaching approximately 20 % in individuals over 75 years old. In fact, in Western countries, HF is regarded as the leading cause of hospitalization among patients older than 65 years. Moreover, some researchers even classify HF as a geriatric syndrome, as it is associated with a poorer prognosis, significant residual disability, and complex comorbidities common in the elderly, which can further complicate the disease's progression [24]. Additionally, although the pharmacokinetics of antibiotics have been demonstrated to be favorably modified in sepsis, numerous studies have shown that antibiotic resistance plays a significant role in elevating mortality rates for most pathogens in septic patients [25]. We are currently entering, and will continue to face, a post-antibiotic era, in which the antibiotics available to us are increasingly ineffective against the emerging bacterial infections [26]. Furthermore, management of patients with both HF and sepsis presents a complex challenge for physicians. Intravenous fluid administration is a critical element of clinical practice guidelines and federally mandated performance benchmarks for managing sepsis and septic shock; however, patients with pre-existing congestive HF are at a higher risk of fluid overload [27,28]. Therefore, the lack of clear guidelines for the therapeutic management of patients with both HF and sepsis presents a dilemma, as interventions that are guideline-compliant for either condition alone often conflict [20]. Thus, together, all these reasons: increasing antibiotic resistance, complex therapeutic management, and the increasing frailty of patients in this age group, can be attributed to the rising mortality associated with concomitant sepsis and HF.

We also studied demographic variations in the mortality trends of our study population. One of the findings is the higher mortality shown by men than women throughout the study period. This disparity can be attributed to the higher mortality rates for HF and sepsis observed in men compared to women when these conditions are considered individually [12,29,30]. Despite significant advancements in understanding sex-based differences in HF in recent years, many aspects remain unclear. Variations in traditional risk factors, along with sex-specific risk factors, uniquely influence the prevalence and presentation of HF [31]. Another study reported that despite substantial efforts to develop new treatment options for sepsis and numerous failed clinical trials, fatality rates for septic shock have remained at 25 %–30 %, and one suggested factor contributing to the unsuccessful translation of therapies for sepsis is sex bias [32]. However, further comprehensive researches are warranted to elaborate on sex and gender-based differences and to develop sex-sensitive strategies and guidelines to improve health outcomes.

Our findings also reveal substantial disparities among racial and ethnic groups, with fatality rates being recorded as the highest in older NH-Black adults, while the lowest in older NH-Asian adults. Racial disparities in sepsis-related mortality are likely attributable to a combination of factors, including variations in environmental conditions and disparities in healthcare access, leading to higher rates of infections and higher risks of organ dysfunctions in some races [33,34]. For instance, Black individuals are more likely to live in medically underserved localities, where communities with higher Black populations often have limited primary care access [35]. It has also been noticed that both Black and Hispanic individuals are increasingly encountering barriers to timely medical care in comparison to their White counterparts [35]. Additionally, with the recent shift in the Centers for Medicare and Medicaid Services' Severe Sepsis/Septic Shock Early Management Bundle from pay-for-reporting to pay-for-performance in 2024 [36], hospitals that mainly serve Black and Hispanic patients may face significant challenges [35]. Furthermore, the highest mortality rates observed in Black individuals for both sepsis and HF, when analyzed separately, may also be a contributing factor [12,30,37].

We also examined the significant geographical variations in the mortality trends. The Southern and Western regions had the highest and lowest mortality, respectively. A report using data from the National Vital Statistics System (NVSS) of the NCHS (2017) identified the top five states with the highest mortality rates as Alabama, Kentucky, Mississippi, Oklahoma, and West Virginia, all of which are located in the Southern region. These states, with the highest overall mortality rates, also reported the highest mortality rates due to heart disease. Additionally, older adults in these states exhibited the highest mortality, which aligns with our findings [38]. Another report analyzing NVSS data from 1973 to 2010, which examined the geographic patterns of heart disease mortality, highlighted a shift in high-mortality counties from the Northeast to the Deep South in a short period of time [39]. In addition to heart failure, other cardiovascular diseases, such as stroke [40] and myocardial infarction [41], also contribute to the highest mortality rates in the Southern region. In fact, due to the exceptionally high death rates, the Southeastern region is frequently referred to as the “stroke belt” [42]. Systematic changes in a range of biomedical, behavioral, and socioenvironmental factors may have contributed to this disparity [39]. Additionally, rural localities had a higher mortality burden compared to urban localities. A study suggests that this trend may be attributed to lower socioeconomic status, alongside the limited number of primary care physicians and cardiologists in those regions [12]. However, there is a scarcity of data that studies regional disparities concerning concomitant HF and sepsis, despite being one of the common causes of morbidity and mortality. Our study also reported that most of the deaths occurred at medical facilities. This is because both sepsis and HF account for significant hospitalizations and inpatient mortality [34,43].

The pathophysiology of sepsis-induced myocardial dysfunction remains undefined, with ongoing discussions encompassing both the underlying mechanisms and treatment strategies [44]. Currently, only supportive treatments are available for patients with sepsis, and no specific medication has been identified that can reverse the myocardial dysfunction associated with sepsis [44]. Future clinical and preclinical studies are heavily required to address this research gap. Targeted multidisciplinary interventions can offer significant benefits to vulnerable subgroups in the management of sepsis and cardiovascular care. Additionally, future studies should examine the risk factors that may be responsible for racial and regional disparities.

### 4.1 Limitations

Nevertheless, our study has certain limitations that need to be considered. We employed ICD-10 codes and used the data from death certificates, which may introduce the potential for misrepresentation of heart failure and sepsis due to possible medical misdiagnoses or human error. The lack of laboratory or imaging data, echocardiographic results, assessment of confounders such as certain sociodemographic factors and comorbidities, and other HF and sepsis-related specifications significantly constrains the depth of our analysis.

### 4.2 Conclusion

In conclusion, older citizens in the US exhibited a rising trend of mortality associated with HF and sepsis from 1999 to 2019. However, demographic and geographical variations in trends were observed with men, non-Hispanic Black or African American individuals, and those living in rural regions, having the highest mortality. Heart failure and sepsis are prevalent complications among older adults and represent significant public health concerns. These patients must receive closely monitored and high-quality care. Moreover, extensive research is essential to assist clinicians in developing evidence-based, algorithmic approaches to optimize treatment strategies for this patient population.

## CRediT authorship contribution statement

**Abdul Ahad:** Conceptualization, Formal analysis, Methodology, Data curation, Investigation, Project administration, Supervision, Visualization, Writing – original draft, Writing – review & editing. **Eeshal Fatima:** Formal analysis, Investigation, Methodology, Writing – original draft, Writing – review & editing. **Adeena Jamil:** Writing – original draft, Writing – review & editing. **Obaid Ur Rehman:** Writing – original draft, Writing – review & editing. **Irfan Ullah:** Supervision, Visualization, Validation, Writing – review & editing. **Raheel Ahmed:** Supervision, Visualization, Validation, Writing – review & editing. **Bernardo Cortese:** Supervision, Validation, Visualization, Writing – review & editing. **Michael E. Hall:** Supervision, Visualization, Validation, Writing – review & editing. **Mamas A. Mamas:** Supervision, Visualization, Validation, Writing – review & editing.

## Declaration

This manuscript represents original research with every author's contribution. All authors declare no conflicts of interest. We agree to all conditions and grant publication rights. The manuscript has been approved for submission to the journal by all the authors.

## Research data

The data utilized to carry out this research study have been obtained from the CDC WONDER database (https://wonder.cdc.gov/). It is government-issued data and can be freely accessed from the internet.

## Funding

This research did not receive any specific grant from funding agencies in the public, commercial, or not-for-profit sectors.

## Declaration of competing interest

The authors declare that they have no known competing financial interests or personal relationships that could have appeared to influence the work reported in this paper.
