# Supplementary material for: Exploring the rising death rates among older US citizens with heart failure and sepsis: Need for health-care policy reform
Source: Int J Cardiol Cardiovasc Risk Prev. 2025 May 11;26:200428. doi: 10.1016/j.ijcrp.2025.200428 (PMC12173663; doi:10.1016/j.ijcrp.2025.200428)
Supplement: Multimedia component 1 [file mmc1.docx]

**Supplemental Table A.1** The absolute number of deaths due to co-existing heart failure and sepsis, categorized according to sex and race, among older adults (65 years and above) in the United States, 1999 to 2019.

| **Year** | **Overall** | **Women** | **Men** | **NH White** | **NH Black or African American** | **Hispanic or Latino** | **NH Asian or Pacific Islander** | **NH American Indian or Alaska Native** | **Population** |
| --- | --- | --- | --- | --- | --- | --- | --- | --- | --- |
| 1999 | 9916 | 5833 | 4083 | 8027 | 1312 | 383 | 138 | 29 | 34797841 |
| 2000 | 9873 | 5837 | 4036 | 7919 | 1348 | 423 | 119 | 39 | 34991753 |
| 2001 | 9841 | 5783 | 4058 | 7875 | 1305 | 451 | 158 | 23 | 35290291 |
| 2002 | 9984 | 5800 | 4184 | 8017 | 1315 | 407 | 169 | 44 | 35522207 |
| 2003 | 10439 | 6035 | 4404 | 8406 | 1324 | 480 | 162 | 43 | 35863529 |
| 2004 | 10617 | 5994 | 4623 | 8551 | 1307 | 516 | 192 | 31 | 36203319 |
| 2005 | 11270 | 6462 | 4808 | 8962 | 1417 | 618 | 212 | 39 | 36649798 |
| 2006 | 11022 | 6203 | 4819 | 8825 | 1321 | 594 | 217 | 46 | 37164107 |
| 2007 | 10387 | 5769 | 4618 | 8240 | 1271 | 606 | 215 | 45 | 37825711 |
| 2008 | 10677 | 5954 | 4723 | 8524 | 1284 | 556 | 240 | 58 | 38777621 |
| 2009 | 10484 | 5606 | 4878 | 8388 | 1229 | 555 | 218 | 79 | 39623175 |
| 2010 | 10683 | 5895 | 4788 | 8517 | 1250 | 595 | 245 | 58 | 40267984 |
| 2011 | 11132 | 6000 | 5132 | 8894 | 1239 | 679 | 253 | 51 | 41394141 |
| 2012 | 10923 | 5770 | 5153 | 8630 | 1275 | 659 | 274 | 59 | 43145356 |
| 2013 | 11651 | 6107 | 5544 | 9150 | 1367 | 732 | 308 | 63 | 44704074 |
| 2014 | 12338 | 6444 | 5894 | 9793 | 1354 | 779 | 299 | 58 | 46243211 |
| 2015 | 13990 | 7210 | 6780 | 11030 | 1574 | 907 | 334 | 85 | 47760852 |
| 2016 | 14629 | 7481 | 7148 | 11564 | 1621 | 964 | 339 | 98 | 49244195 |
| 2017 | 15952 | 8047 | 7905 | 12386 | 1824 | 1143 | 452 | 107 | 50858679 |
| 2018 | 16912 | 8412 | 8500 | 13353 | 1860 | 1111 | 441 | 112 | 52431193 |
| 2019 | 17395 | 8432 | 8963 | 13645 | 1941 | 1217 | 451 | 101 | 54058263 |
| **Total** | **250115** | **135074** | **115041** | **198696** | **29738** | **14375** | **5436** | **1268** | **872817300** |

NH =non-Hispanic

**Supplemental Table A.2** The absolute number of deaths due to co-existing heart failure and sepsis, categorized according to location of death, among older adults (65 years and above) in the United States, 1999 to 2019.

| **Year** | **Medical Facility** | **Nursing Home/Long-term Care Facility** | **Residences** | **Hospices** |  |
| --- | --- | --- | --- | --- | --- |
|  |  |  |  |  |  |
| 1999 | 8275 | 1337 | 221 | Missing |  |
| 2000 | 8261 | 1298 | 236 | Missing |  |
| 2001 | 8292 | 1225 | 223 | Missing |  |
| 2002 | 8440 | 1199 | 235 | Missing |  |
| 2003 | 8827 | 1196 | 251 | 10 |  |
| 2004 | 8919 | 1191 | 288 | 25 |  |
| 2005 | 9426 | 1253 | 321 | 56 |  |
| 2006 | 9293 | 1118 | 353 | 97 |  |
| 2007 | 8750 | 1047 | 308 | 144 |  |
| 2008 | 8946 | 980 | 311 | 181 |  |
| 2009 | 8573 | 974 | 329 | 221 |  |
| 2010 | 8841 | 1017 | 352 | 301 |  |
| 2011 | 9049 | 1088 | 437 | 357 |  |
| 2012 | 8774 | 1046 | 430 | 478 |  |
| 2013 | 9400 | 1019 | 519 | 471 |  |
| 2014 | 9868 | 1082 | 576 | 646 |  |
| 2015 | 11075 | 1236 | 651 | 895 |  |
| 2016 | 11420 | 1229 | 772 | 1050 |  |
| 2017 | 12352 | 1384 | 842 | 1203 |  |
| 2018 | 13030 | 1418 | 939 | 1341 |  |
| 2019 | 13454 | 1376 | 889 | 1471 |  |
| **Total** | **203368** | **24713** | **9483** | **8947** |  |

**Supplemental Table A.3** Age-adjusted overall mortality rate, per 100,000 population, for co-existing heart failure and sepsis, heart failure alone, and sepsis alone, among older adults (65 years and above) in the United States, 1999 to 2019.

| **Age-Adjusted Mortality Rate (95% CI)** | | | |
| --- | --- | --- | --- |
| **Year** | **Co-existing Heart Failure and Sepsis** | **Heart Failure alone** | **Sepsis alone** |
| 1999 | 28.8 (28.3 - 29.4) | 793.1 (790.1-796.1) | 306.0 (304.1-307.8) |
| 2000 | 28.4 (27.8 - 29.0) | 782.7 (779.8-785.6) | 300.2 (298.3-302.0) |
| 2001 | 27.9 (27.3 - 28.4) | 763.2 (760.3-766.1) | 299.9 (298.1-301.7) |
| 2002 | 28.0 (27.4 - 28.5) | 749.1 (746.3-751.9) | 303.3 (301.5-305.1) |
| 2003 | 28.9 (28.3 - 29.4) | 742.7 (739.9-745.5) | 301.2 (299.4-303.0) |
| 2004 | 29.0 (28.5 - 29.6) | 718.7 (715.9-721.4) | 297.4 (295.6-299.1) |
| 2005 | 30.2 (29.7 - 30.8) | 723.3 (720.5-726.0) | 306.9 (305.1-308.7) |
| 2006 | 29.0 (28.5 - 29.6) | 682.5 (679.9-685.1) | 299.4 (297.6-301.1) |
| 2007 | 26.9 (26.4 - 27.4) | 654.6 (652.1-657.1) | 293.6 (291.9-295.4) |
| 2008 | 27.1 (26.5 - 27.6) | 650.5 (648.0-653.0) | 299.9 (298.2-301.7) |
| 2009 | 26.1 (25.6 - 26.6) | 620.1 (617.6-622.5) | 288.3 (286.6-290.0) |
| 2010 | 26.2 (25.7 - 26.7) | 620.5 (618.1-622.9) | 289.8 (288.1-291.4) |
| 2011 | 26.5 (26.0 - 27.0) | 611.7 (609.4-614.1) | 289.7 (288.0-291.3) |
| 2012 | 25.2 (24.8 - 25.7) | 598.6 (596.3-600.9) | 282.9 (281.3-284.5) |
| 2013 | 26.3 (25.8 - 26.8) | 611.3 (609.0-613.6) | 289.8 (288.2-291.4) |
| 2014 | 27.1 (26.6 - 27.6) | 614.3 (612.0-616.6) | 294.8 (293.2-296.4) |
| 2015 | 30.0 (29.5 - 30.5) | 642.9 (640.6-645.2) | 310.7 (309.1-312.3) |
| 2016 | 30.7 (30.2 - 31.2) | 640.0 (637.7-642.3) | 305.7 (304.1-307.3) |
| 2017 | 32.4 (31.9 - 33.0) | 659.1 (656.8-661.4) | 308.7 (307.2-310.3) |
| 2018 | 33.6 (33.1 - 34.1) | 671.1 (668.8-673.4) | 303.6 (302.0-305.1) |
| 2019 | 33.7 (33.2 - 34.2) | 680.6 (678.4-682.9) | 288.5 (287.0-290.0) |
| **Total** | **28.8 (28.7 - 29.0)** | **673.0 (672.4-673.5)** | **298.1 (297.7-298.4)** |

CI = confidence interval

**Supplemental Table A.4** Age-adjusted mortality rate, per 100,000 population, for co-existing heart failure and sepsis, categorized according to sex, among older adults (65 years and above) in the United States, 1999 to 2019.

| **Age-Adjusted Mortality Rate (95% CI)** | | |
| --- | --- | --- |
| **Year** | **Men** | **Women** |
| 1999 | 32.6 (31.6-33.6) | 26.6 (25.9-27.3) |
| 2000 | 31.5 (30.5-32.5) | 26.3 (25.7-27.0) |
| 2001 | 31.3 (30.3-32.2) | 25.8 (25.2-26.5) |
| 2002 | 31.6 (30.6-32.6) | 25.7 (25.0-26.3) |
| 2003 | 32.6 (31.6-33.5) | 26.6 (25.9-27.2) |
| 2004 | 33.7 (32.7-34.6) | 26.2 (25.6-26.9) |
| 2005 | 34.1 (33.2-35.1) | 27.8 (27.1-28.5) |
| 2006 | 33.4 (32.5-34.4) | 26.3 (25.6-26.9) |
| 2007 | 31.2 (30.3-32.1) | 24.1 (23.5-24.8) |
| 2008 | 31.1 (30.2-32.0) | 24.4 (23.8-25.1) |
| 2009 | 31.2 (30.3-32.1) | 22.8 (22.2-23.4) |
| 2010 | 30.2 (29.3-31.0) | 23.6 (23.0-24.2) |
| 2011 | 31.2 (30.3-32.0) | 23.4 (22.8-24.0) |
| 2012 | 30.2 (29.4-31.0) | 21.9 (21.4-22.5) |
| 2013 | 31.3 (30.5-32.2) | 22.9 (22.3-23.5) |
| 2014 | 32.2 (31.4-33.0) | 23.7 (23.1-24.3) |
| 2015 | 35.9 (35.0-36.7) | 26.0 (25.4-26.6) |
| 2016 | 36.7 (35.9-37.6) | 26.4 (25.8-27.0) |
| 2017 | 39.2 (38.4-40.1) | 27.7 (27.1-28.3) |
| 2018 | 41.0 (40.1-41.9) | 28.3 (27.7-28.9) |
| 2019 | 41.8 (40.9-42.7) | 28.0 (27.4-28.6) |
| **Total** | **33.9 (33.7-34.1)** | **25.5 (25.3-25.6)** |

CI = confidence interval

**Supplemental Table A.5** Age-adjusted mortality rate, per 100,000 population, for co-existing heart failure and sepsis, categorized according to race and ethnicity, among older (65 years and above) adults in the United States, 1999 to 2019.

| **Age-Adjusted Mortality Rate (95% CI)** | | | | | |
| --- | --- | --- | --- | --- | --- |
| **Year** | **NH Black or African American** | **NH American Indian or Alaska Native** | **NH White** | **Hispanic or Latino** | **NH Asian or Pacific Islander** |
| 1999 | 49.3 (46.7-52.0) | 26.3 (17.5-38.0) | 27.1 (26.5-27.7) | 27.0 (24.3-29.8) | 21.3 (17.6-24.9) |
| 2000 | 50.4 (47.7-53.1) | 32.5 (23.0-44.7) | 26.6 (26.0-27.1) | 28.5 (25.7-31.2) | 16.7 (13.7-19.8) |
| 2001 | 48.2 (45.6-50.8) | 18.0 (11.3-27.3) | 26.1 (25.5-26.7) | 28.7 (26.0-31.4) | 20.6 (17.3-23.9) |
| 2002 | 48.4 (45.8-51.0) | 33.9 (24.4-45.8) | 26.4 (25.8-27.0) | 24.7 (22.3-27.2) | 20.9 (17.7-24.1) |
| 2003 | 48.1 (45.5-50.7) | 32.7 (23.5-44.4) | 27.4 (26.8-27.9) | 27.4 (24.9-29.8) | 18.4 (15.5-21.3) |
| 2004 | 46.6 (44.1-49.2) | 23.9 (16.1-34.1) | 27.7 (27.1-28.3) | 27.9 (25.5-30.4) | 21.9 (18.7-25.0) |
| 2005 | 49.5 (46.9-52.1) | 27.6 (19.4-38.0) | 28.6 (28.0-29.2) | 32.1 (29.6-34.7) | 21.5 (18.6-24.5) |
| 2006 | 45.3 (42.9-47.8) | 30.3 (22.1-40.7) | 27.7 (27.1-28.3) | 29.2 (26.8-31.6) | 21.5 (18.6-24.4) |
| 2007 | 42.5 (40.2-44.9) | 27.7 (20.0-37.5) | 25.6 (25.0-26.1) | 28.6 (26.3-30.9) | 19.5 (16.9-22.1) |
| 2008 | 41.9 (39.6-44.3) | 37.1 (28.0-48.1) | 26.0 (25.4-26.5) | 24.8 (22.7-26.9) | 20.8 (18.1-23.4) |
| 2009 | 38.7 (36.6-40.9) | 49.1 (38.6-61.5) | 25.2 (24.7-25.8) | 23.1 (21.1-25.0) | 17.4 (15.0-19.7) |
| 2010 | 38.8 (36.6-41.0) | 34.4 (25.9-44.7) | 25.3 (24.7-25.8) | 24.1 (22.1-26.0) | 18.9 (16.5-21.3) |
| 2011 | 37.2 (35.1-39.3) | 28.5 (21.0-37.8) | 25.8 (25.3-26.3) | 25.3 (23.4-27.2) | 17.9 (15.7-20.2) |
| 2012 | 36.6 (34.6-38.6) | 32.6 (24.7-42.2) | 24.5 (24.0-25.0) | 22.9 (21.1-24.6) | 18.1 (16.0-20.3) |
| 2013 | 37.5 (35.5-39.5) | 32.2 (24.6-41.4) | 25.5 (25.0-26.1) | 23.9 (22.2-25.7) | 18.6 (16.5-20.7) |
| 2014 | 35.7 (33.8-37.6) | 26.1 (19.7-34.0) | 26.8 (26.3-27.4) | 23.8 (22.1-25.5) | 16.8 (14.9-18.7) |
| 2015 | 40.2 (38.1-42.2) | 37.5 (29.8-46.6) | 29.7 (29.1-30.2) | 26.2 (24.5-28.0) | 17.4 (15.5-19.3) |
| 2016 | 39.5 (37.6-41.5) | 40.5 (32.7-49.5) | 30.6 (30.1-31.2) | 26.3 (24.7-28.0) | 16.8 (15.0-18.6) |
| 2017 | 42.8 (40.8-44.8) | 40.5 (32.7-48.4) | 32.0 (31.5-32.6) | 29.5 (27.7-31.2) | 20.7 (18.8-22.7) |
| 2018 | 42.0 (40.1-43.9) | 40.0 (32.4-47.6) | 33.9 (33.3-34.5) | 27.5 (25.9-29.2) | 19.1 (17.3-20.9) |
| 2019 | 41.6 (39.7-43.5) | 34.9 (28.0-41.9) | 34.0 (33.5-34.6) | 28.8 (27.2-30.4) | 18.5 (16.7-20.2) |
| **Total** | **42.4 (41.9-42.9)** | **33.6 (31.7-35.5)** | **27.9 (27.8-28.0)** | **26.5 (26.1-27.0)** | **18.9 (18.4-19.5)** |

NH = non-Hispanic; CI = confidence interval

**Supplemental Table A.6** Age-adjusted mortality rate, per 100,000 population, for co-existing heart failure and sepsis, categorized according to states, among older adults (65 years and above) in the United States, 1999 to 2019.

| **State** | **Age-Adjusted Mortality Rate (95% CI)** | **Rank** | **Percentile** |
| --- | --- | --- | --- |
| Mississippi | 45.4 (43.9-46.8) | 1 | 100.00% |
| West Virginia | 45.0 (43.4-46.7) | 2 | 98.00% |
| Oklahoma | 44.7 (43.4-45.9) | 3 | 96.00% |
| Kentucky | 42.7 (41.5-43.9) | 4 | 94.00% |
| District of Columbia | 41.8 (38.5-45.0) | 5 | 92.00% |
| Rhode Island | 41.7 (39.6-43.9) | 6 | 90.00% |
| Texas | 40.6 (40.0-41.1) | 7 | 88.00% |
| New Jersey | 37.7 (37.0-38.5) | 8 | 86.00% |
| Arkansas | 36.4 (35.1-37.7) | 9 | 84.00% |
| Alabama | 34.9 (33.9-35.9) | 10 | 82.00% |
| South Carolina | 34.8 (33.7-35.8) | 11 | 80.00% |
| Tennessee | 33.2 (32.4-34.1) | 12 | 78.00% |
| Louisiana | 32.0 (30.9-33.0) | 13 | 76.00% |
| Indiana | 31.8 (30.9-32.6) | 14 | 74.00% |
| Ohio | 31.7 (31.1-32.3) | 15 | 72.00% |
| Maryland | 31.5 (30.6-32.4) | 16 | 70.00% |
| North Carolina | 31.2 (30.5-31.9) | 17 | 68.00% |
| California | 31.1 (30.7-31.4) | 18 | 66.00% |
| Connecticut | 30.9 (29.9-31.9) | 19 | 64.00% |
| Illinois | 30.1 (29.5-30.6) | 20 | 62.00% |
| Michigan | 29.5 (28.8-30.1) | 21 | 60.00% |
| Massachusetts | 29.3 (28.5-30.0) | 22 | 58.00% |
| Washington | 29.2 (28.4-30.0) | 23 | 56.00% |
| Pennsylvania | 28.2 (27.7-28.7) | 24 | 54.00% |
| New York | 27.7 (27.2-28.1) | 25 | 52.00% |
| Virginia | 27.3 (26.5-28.0) | 26 | 50.00% |
| Delaware | 26.4 (24.4-28.3) | 27 | 48.00% |
| Georgia | 26.3 (25.6-27.0) | 28 | 46.00% |
| South Dakota | 24.9 (23.1-26.8) | 29 | 44.00% |
| Missouri | 24.3 (23.5-25.0) | 30 | 42.00% |
| North Dakota | 23.9 (21.9-25.9) | 31 | 40.00% |
| Kansas | 23.8 (22.7-24.8) | 32 | 38.00% |
| New Hampshire | 22.8 (21.3-24.3) | 33 | 36.00% |
| Nebraska | 22.2 (20.9-23.4) | 34 | 34.00% |
| Iowa | 21.9 (21.0-22.8) | 35 | 32.00% |
| Utah | 21.8 (20.5-23.1) | 36 | 30.00% |
| Vermont | 21.7 (19.7-23.8) | 37 | 28.00% |
| Maine | 21.6 (20.2-22.9) | 38 | 26.00% |
| Alaska | 21.4 (18.4-24.4) | 39 | 24.00% |
| Nevada | 21.3 (20.2-22.5) | 40 | 22.00% |
| Oregon | 20.9 (20.1-21.8) | 41 | 20.00% |
| Minnesota | 20.6 (19.9-21.4) | 42 | 16.00% |
| Wisconsin | 20.6 (19.9-21.2) | 42 | 16.00% |
| Idaho | 20.1 (18.7-21.5) | 44 | 12.00% |
| New Mexico | 20.1 (18.9-21.3) | 44 | 12.00% |
| Colorado | 19.6 (18.8-20.4) | 46 | 10.00% |
| Wyoming | 19.4 (17.1-21.6) | 47 | 8.00% |
| Montana | 18.4 (16.9-19.9) | 48 | 6.00% |
| Hawaii | 17.1 (15.9-18.4) | 49 | 4.00% |
| Florida | 16.8 (16.5-17.1) | 50 | 2.00% |
| Arizona | 12.1 (11.6-12.6) | 51 | 0.00% |

CI = confidence interval

**Supplemental Table A.7** Age-adjusted mortality rate, per 100,000 populations, for co-existing heart failure and sepsis, categorized according to region, among older adults (65 years and above) in the United States, 1999 to 2019.

| **Region** | **Year** | **Age-Adjusted Mortality Rate (95% CI)** |
| --- | --- | --- |
| South | 1999 | 31.9 (30.9-32.9) |
| South | 2000 | 31.1 (30.1-32.1) |
| South | 2001 | 30.7 (29.7-31.7) |
| South | 2002 | 31.2 (30.2-32.2) |
| South | 2003 | 32.8 (31.8-33.8) |
| South | 2004 | 31.2 (30.2-32.1) |
| South | 2005 | 33.0 (32.0-34.0) |
| South | 2006 | 31.3 (30.4-32.3) |
| South | 2007 | 29.4 (28.5-30.3) |
| South | 2008 | 28.9 (28.0-29.8) |
| South | 2009 | 28.6 (27.7-29.4) |
| South | 2010 | 27.9 (27.1-28.8) |
| South | 2011 | 27.0 (26.1-27.8) |
| South | 2012 | 26.3 (25.4-27.1) |
| South | 2013 | 27.5 (26.7-28.3) |
| South | 2014 | 28.2 (27.4-29.0) |
| South | 2015 | 31.7 (30.8-32.5) |
| South | 2016 | 32.3 (31.4-33.1) |
| South | 2017 | 35.0 (34.2-35.9) |
| South | 2018 | 36.6 (35.7-37.4) |
| South | 2019 | 36.4 (35.6-37.3) |
| **South** | **Total** | **31.1 (30.9-31.3)** |
| Northeast | 1999 | 32.3 (31.0-33.6) |
| Northeast | 2000 | 33.0 (31.7-34.3) |
| Northeast | 2001 | 30.9 (29.7-32.2) |
| Northeast | 2002 | 30.1 (28.9-31.3) |
| Northeast | 2003 | 30.0 (28.8-31.2) |
| Northeast | 2004 | 31.8 (30.5-33.0) |
| Northeast | 2005 | 32.4 (31.1-33.7) |
| Northeast | 2006 | 31.2 (30.0-32.4) |
| Northeast | 2007 | 27.5 (26.4-28.7) |
| Northeast | 2008 | 28.3 (27.2-29.5) |
| Northeast | 2009 | 25.4 (24.3-26.5) |
| Northeast | 2010 | 27.9 (26.7-29.0) |
| Northeast | 2011 | 27.7 (26.5-28.8) |
| Northeast | 2012 | 26.6 (25.5-27.7) |
| Northeast | 2013 | 27.9 (26.7-29.0) |
| Northeast | 2014 | 29.7 (28.5-30.8) |
| Northeast | 2015 | 30.0 (28.9-31.1) |
| Northeast | 2016 | 29.8 (28.7-31.0) |
| Northeast | 2017 | 29.8 (28.6-30.9) |
| Northeast | 2018 | 30.8 (29.7-32.0) |
| Northeast | 2019 | 30.8 (29.7-31.9) |
| **Northeast** | **Total** | **29.7 (29.4-29.9)** |
| Midwest | 1999 | 27.4 (26.3-28.6) |
| Midwest | 2000 | 26.7 (25.6-27.8) |
| Midwest | 2001 | 26.4 (25.3-27.5) |
| Midwest | 2002 | 26.7 (25.6-27.8) |
| Midwest | 2003 | 27.8 (26.7-28.9) |
| Midwest | 2004 | 27.5 (26.4-28.6) |
| Midwest | 2005 | 28.3 (27.2-29.4) |
| Midwest | 2006 | 26.7 (25.7-27.8) |
| Midwest | 2007 | 25.0 (24.0-26.1) |
| Midwest | 2008 | 25.7 (24.7-26.8) |
| Midwest | 2009 | 25.0 (24.0-26.0) |
| Midwest | 2010 | 23.8 (22.8-24.8) |
| Midwest | 2011 | 25.6 (24.6-26.6) |
| Midwest | 2012 | 24.0 (23.0-25.0) |
| Midwest | 2013 | 23.6 (22.6-24.5) |
| Midwest | 2014 | 25.7 (24.7-26.7) |
| Midwest | 2015 | 29.1 (28.0-30.1) |
| Midwest | 2016 | 29.3 (28.3-30.4) |
| Midwest | 2017 | 30.7 (29.6-31.8) |
| Midwest | 2018 | 32.5 (31.5-33.6) |
| Midwest | 2019 | 32.9 (31.9-34.0) |
| **Midwest** | **Total** | **27.3 (27.1-27.5)** |
| West | 1999 | 21.3 (20.2-22.4) |
| West | 2000 | 20.5 (19.4-21.5) |
| West | 2001 | 21.5 (20.4-22.6) |
| West | 2002 | 21.6 (20.5-22.7) |
| West | 2003 | 22.1 (21.0-23.2) |
| West | 2004 | 24.1 (23.0-25.2) |
| West | 2005 | 25.4 (24.3-26.6) |
| West | 2006 | 25.6 (24.4-26.7) |
| West | 2007 | 23.8 (22.8-24.9) |
| West | 2008 | 24.1 (23.1-25.2) |
| West | 2009 | 23.8 (22.7-24.8) |
| West | 2010 | 24.1 (23.1-25.2) |
| West | 2011 | 25.3 (24.3-26.4) |
| West | 2012 | 23.5 (22.6-24.5) |
| West | 2013 | 25.5 (24.5-26.5) |
| West | 2014 | 24.4 (23.4-25.4) |
| West | 2015 | 27.7 (26.7-28.8) |
| West | 2016 | 29.9 (28.8-31.0) |
| West | 2017 | 32.2 (31.1-33.3) |
| West | 2018 | 31.7 (30.7-32.8) |
| West | 2019 | 32.1 (31.1-33.2) |
| **West** | **Total** | **25.8 (25.6-26.0)** |

CI = confidence interval

**Supplemental Table A.8** Age-adjusted mortality rate, per 100,000 populations, for co-existing heart failure and sepsis, classified according to urbanization, among older adults (65 years and above) in the United States, 1999 to 2019.

| **Age-Adjusted Mortality Rate (95% CI)** | | |
| --- | --- | --- |
| **Year** | **Non-metropolitan areas** | **Metropolitan areas** |
| 1999 | 30.0 (28.7-31.3) | 28.6 (28.0-29.2) |
| 2000 | 28.9 (27.6-30.2) | 28.2 (27.6-28.9) |
| 2001 | 29.1 (27.8-30.3) | 27.6 (27.0-28.2) |
| 2002 | 28.6 (27.3-29.9) | 27.8 (27.2-28.4) |
| 2003 | 30.2 (28.9-31.5) | 28.5 (27.9-29.1) |
| 2004 | 29.7 (28.5-31.0) | 28.8 (28.2-29.4) |
| 2005 | 30.6 (29.3-31.9) | 30.2 (29.6-30.8) |
| 2006 | 30.8 (29.5-32.1) | 28.7 (28.1-29.3) |
| 2007 | 29.4 (28.1-30.6) | 26.3 (25.8-26.9) |
| 2008 | 27.8 (26.5-29.0) | 26.9 (26.3-27.5) |
| 2009 | 29.5 (28.3-30.8) | 25.4 (24.8-25.9) |
| 2010 | 29.0 (27.8-30.2) | 25.6 (25.0-26.1) |
| 2011 | 28.4 (27.1-29.6) | 26.0 (25.5-26.6) |
| 2012 | 27.1 (25.9-28.3) | 24.9 (24.3-25.4) |
| 2013 | 28.9 (27.7-30.1) | 25.7 (25.2-26.2) |
| 2014 | 30.7 (29.4-31.9) | 26.4 (25.8-26.9) |
| 2015 | 33.8 (32.5-35.1) | 29.2 (28.7-29.7) |
| 2016 | 35.7 (34.4-37.0) | 29.6 (29.1-30.2) |
| 2017 | 37.1 (35.8-38.4) | 31.5 (30.9-32.1) |
| 2018 | 38.9 (37.5-40.2) | 32.5 (31.9-33.0) |
| 2019 | 40.4 (39.1-41.7) | 32.3 (31.8-32.9) |
| **Total** | **31.4 (31.1-31.7)** | **28.3 (28.1-28.4)** |

CI = confidence interval

**Supplemental Table A.9** Annual percent change values of co-existing heart failure and sepsis-associated age-adjusted mortality rates per 100,000 populations, among older adults (65 years and above) in the United States, 1999 to 2019.

| **Year Interval** | **APC value (95% CI)** |
| --- | --- |
| **Co-existing Heart Failure and Sepsis** | |
| **Overall** | |
| 1999 – 2005 | 0.57 (-0.94 to 5.03) |
| 2005 – 2012 | -2.06 (-6.85 to 2.91) |
| 2012 – 2019 | 4.72* (3.20 to 7.37) |
| **Men** | |
| 1999 – 2005 | 0.88 (-0.64 to 6.83) |
| 2005 – 2012 | -1.46 (-5.93 to 6.57) |
| 2012 – 2019 | 5.17* (3.08 to 7.97) |
| **Women** | |
| 1999 – 2005 | 0.28 (-0.89 to 3.48) |
| 2005 – 2012 | -2.50* (-6.24 to -1.33) |
| 2012 – 2019 | 3.97* (2.68 to 5.91) |
| **NH Black or African American** | |
| 1999 – 2005 | -0.64 (-1.87 to 2.46) |
| 2005 – 2011 | -4.50* (-8.38 to -3.02) |
| 2011 – 2019 | 2.09* (1.10 to 3.52) |
| **NH American Indian or Alaska Native** | |
| 1999 – 2019 | 1.63* (0.23 to 3.49) |
| **NH White** | |
| 1999 – 2005 | 0.78 (-0.57 to 4.69) |
| 2005 – 2012 | -1.64 (-5.84 to 3.81) |
| 2012 – 2019 | 5.19* (3.66 to 7.57) |
| **Hispanic or Latino** | |
| 1999 – 2006 | 1.80 (-0.17 to 8.48) |
| 2006 – 2009 | -8.92* (-12.10 to -1.96) |
| 2009 – 2019 | 2.34* (1.23 to 5.04) |
| **NH Asian or Pacific Islander** | |
| 1999 – 2019 | -0.59 (-1.31 to 0.33) |
| **Non-metropolitan areas** | |
| 1999 – 2012 | -0.45 (-1.16 to 0.15) |
| 2012 – 2019 | 5.41* (4.09 to 7.38) |
| **Metropolitan area** | |
| 1999 – 2005 | 0.55 (-0.86 to 4.45) |
| 2005 – 2012 | -2.16 (-6.65 to 1.81) |
| 2012 – 2019 | 4.40* (2.89 to 7.02) |
| **Heart Failure alone - Overall** | |
| 1999 – 2012 | -2.27* (-2.47 to -2.08) |
| 2012 – 2019 | 2.00* (1.49 to 2.61) |
| **Sepsis alone – Overall** | |
| 1999 – 2012 | -0.48* (-0.85 to -0.25) |
| 2012 – 2017 | 1.66* (0.90 to 3.36) |
| 2017 – 2019 | -3.67* (-5.79 to -1.23) |

APC = annual percent change; NH = non-Hispanic; CI = confidence interval

* Denotes that the APC value is significantly different from zero at α = 0.05.
